# Supplementary material for: Active-feedback quantum control of an integrated low-frequency mechanical resonator
Source: Nat Commun. 2023 Aug 5;14:4721. doi: 10.1038/s41467-023-40442-3 (PMC10404274; doi:10.1038/s41467-023-40442-3)
Supplement: Supplementary file 1 — Supplementary Information [file 41467_2023_40442_MOESM1_ESM.pdf]

Supplementary Information
Active-feedback quantum control of an integrated low-frequency mechanical resonator

Jingkun Guo,[1] Jin Chang,[1] Xiong Yao,[1,2,3] and Simon Gröblacher[1,*]

[1] *Kavli Institute of Nanoscience, Department of Quantum Nanoscience, Delft University of Technology, 2628CJ Delft, The Netherlands*
[2] *Faculty of Physics, School of Science, Westlake University, Hangzhou 310030, P.R. China*
[3] *Department of Physics, Fudan University, Shanghai 200438, P.R. China*

EXPERIMENTAL PARAMETERS

The measured experimental parameters are listed in the following table.

**Parameters**	**Liquid helium, with heterodyne (Fig. 2)**	**Liquid helium, without heterodyne (Fig. 3)**	**Liquid nitrogen, with heterodyne (Fig. 4)**
Temperature under sample stage (T)	6 K		77 K
Mechanical frequency (Ω_{M})	1.045 MHz		1.034 MHz
Effective mass	16 pg		
Mechanical quality factor (Q_{M})	5.1×10^7		4.1×10^7
Optical cavity linewidth ($\kappa/2\pi$)	8.8 GHz		
Optical cavity external coupling rate ($\kappa_{\mathrm{e}}/2\pi$)	6.9 GHz		
Optomechanical coupling rate ($g_0/2\pi$)	224 kHz		
Input power for cooling	0.79 μW	0.76 μW	0.53 μW
Input power for heterodyne detection	1.3 μW	N/A	1.6 μW
Cooling detection efficiency (excl. $\kappa_{\mathrm{e}}/\kappa$)	42%	49%	37%
Efficiency from the input of the circulator to the input of photodetector	59%	69%	60%
Fiber coupling efficiency	91%	90%	78%
Photodetector quantum efficiency	79%		

TABLE S1. Experimental parameters.

LASER NOISE FOR SIDEBAND-ASYMMETRY DETECTION

The phase and amplitude noise of the heterodyne detection laser affects the observed sideband asymmetry [1]. We verify that they do not have significant effect on the heterodyne detection.

Classical laser amplitude noise might enhance the asymmetry [1]. We measure the laser amplitude noise by sending light to a variable coupler (beamsplitter) and then a balanced photodetector, as shown in Figure S1(a). We then take the spectrum of the received signal. When the splitting ratio of the variable coupler is set to 50:50, the classical noise is canceled, and the spectrum of the measured signal shows the amplitude shot noise, and additional electronic noise. When the variable coupler is set to 100:0, the measured spectrum consists of classical laser noise, shot noise, and electronic noise. The electronic noise is measured by blocking the laser. After subtracting the electronic noise, we can estimate the ratio of the classical amplitude noise to the shot noise. For our typical measurement power (~ 1.5 μW), excess classical noise, normalized to shot noise, is about 2×10^{-3}. The measured spectrum and the classical amplitude noise are shown in Figure S1(b,c).

* s.groeblacher@tudelft.nl

The impact of the classical phase noise to the laser, whose PSD is C_Y when normalized to the shot noise, is approximately $4(\Delta\omega/\kappa^2)C_\mathrm{Y}$ [1]. The additional noise is added to both sidebands [1], reducing the sideband asymmetery. The laser phase noise is measured by sending the laser through a filter cavity. The laser is on resonance with the filter cavity, and we do a phase measurement through a homodyne setup (Figure S1(d)). The measured spectrum is compared to a phase calibration tone (1.05 MHz) generated by a phase EOM at the common path of the homodyne setup, and it allows us to calibrate the phase noise PSD C_θ. We then calculate the normalized phase quadrature spectrum by using the input power to the filter cavity [1], $C_\mathrm{Y} = 2nC_\theta$, where n is the input photon flux. The classical phase noise around the mechanical frequency $C_\mathrm{Y} \approx 430$, and the correction due to the classical laser phase noise is $4(\Delta\omega/\kappa^2)C_\mathrm{Y} \approx 0.05$. The measured spectrum and the calculated classical phase noise are shown in Figure S1(e,f).

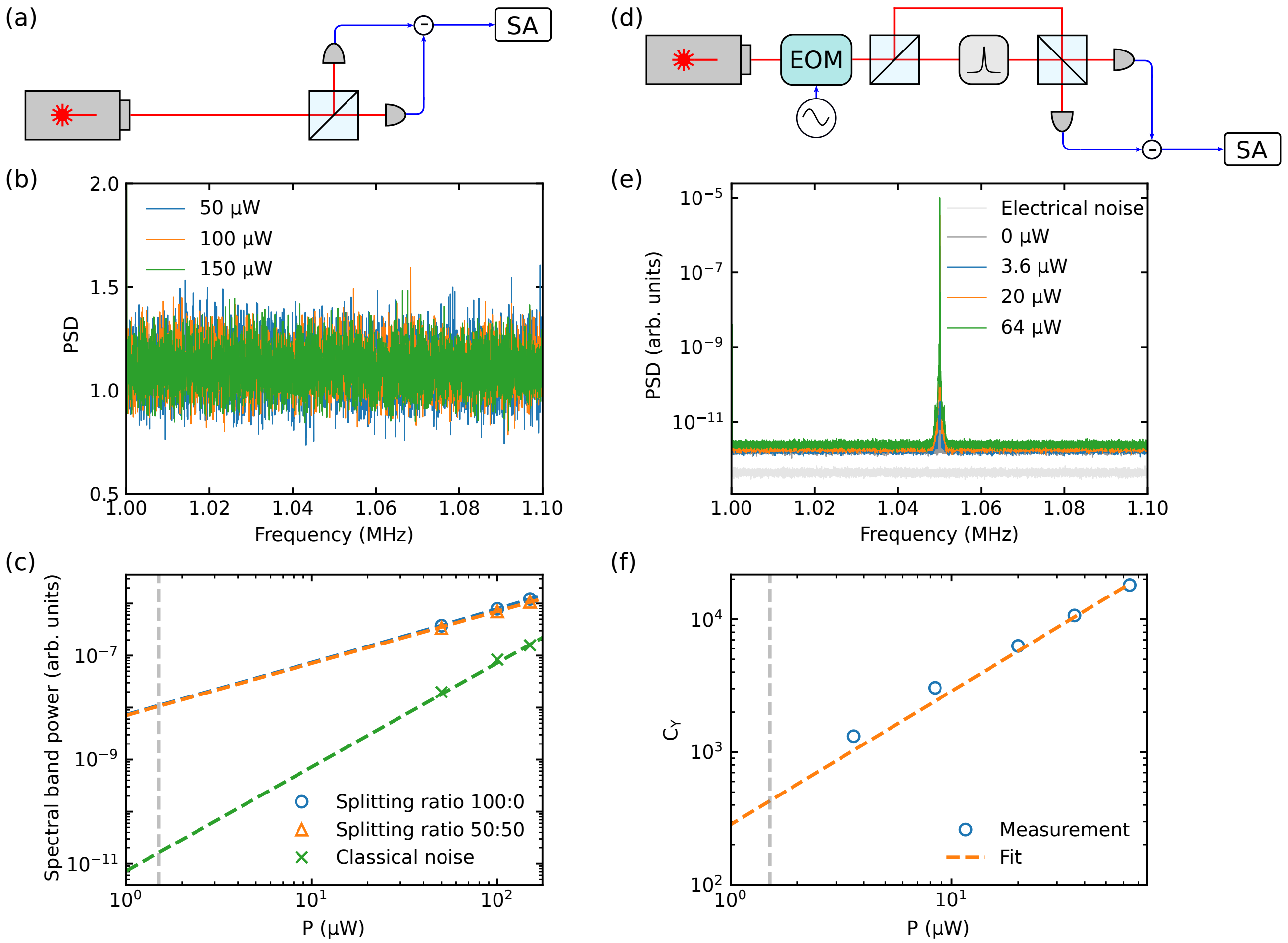

FIG. S1. (a) Laser amplitude noise measurement scheme. The splitting ratio of the variable coupler changes between 50:50 and 100:0 to measure shot noise and total noise. (b) Measured spectrum (normalized to shot noise) when the splitting ratio of the variable coupler is set to 100:0. (c) Measured noise power over a frequency band between 1.02 MHz and 1.08 MHz. The gray dashed line marks the typical heterodyne cavity input power (1.5 µW). The excess classical amplitude noise, when normalized to the shot noise, is about 2×10^{-3}. (d) Laser phase noise measurement scheme and (e) measured spectrum at different cavity input power. (f) C_Y, the classical noise of the phase quadrature normalized to shot noise, as a function of input power, and a linear fit. At typical heterodyne power (1.5 µW, gray dashed line), the excess classical phase noise is about 430. In (b), (c) and (f), the electronic noise is subtracted.

[1] V. Sudhir, D. J. Wilson, R. Schilling, H. Schütz, S. A. Fedorov, A. H. Ghadimi, A. Nunnenkamp, and T. J. Kippenberg, Appearance and Disappearance of Quantum Correlations in Measurement-Based Feedback Control of a Mechanical Oscillator, Phys. Rev. X **7**, 011001 (2017).
